# The use of on-animal acoustical recording devices for studying animal behavior

**DOI:** 10.1002/ece3.608

**Published:** 2013-05-31

**Authors:** Emma Lynch, Lisa Angeloni, Kurt Fristrup, Damon Joyce, George Wittemyer

**Affiliations:** 1 Graduate Degree Program in Ecology, Colorado State UniversityFort Collins, CO, 80523-1474; 2 Department of Biology and Graduate Degree Program in Ecology, Colorado State UniversityFort Collins, CO, 80523-1878; 3 Natural Sounds and Night Skies Division, National Park ServiceFort Collins, CO, 80525; 4 Natural Sounds and Night Skies Division, National Park ServiceFort Collins, CO, 80525; 5 Department of Fish, Wildlife and Conservation Biology and Graduate Degree Program in Ecology, Colorado State UniversityFort Collins, CO, 80523-1474

**Keywords:** Acoustic monitoring, mule deer, sound recording, wildlife behaviour

## Abstract

Audio recordings made from free-ranging animals can be used to investigate aspects of physiology, behavior, and ecology through acoustic signal processing. On-animal acoustical monitoring applications allow continuous remote data collection, and can serve to address questions across temporal and spatial scales. We report on the design of an inexpensive collar-mounted recording device and present data on the activity budget of wild mule deer (*Odocoileus hemionus*) derived from these devices applied for a 2-week period. Over 3300 h of acoustical recordings were collected from 10 deer on their winter range in a natural gas extraction field in northwestern Colorado. Analysis of a subset of the data indicated deer spent approximately 33.5% of their time browsing, 20.8% of their time processing food through mastication, and nearly 38.3% of their time digesting through rumination, with marked differences in diel patterning of these activities. Systematic auditory vigilance was a salient activity when masticating, and these data offer options for quantifying wildlife responses to varying listening conditions and predation risk. These results (validated using direct observation) demonstrate that acoustical monitoring is a viable and accurate method for characterizing individual time budgets and behaviors of ungulates, and may provide new insight into the ways external forces affect wildlife behavior.

## Introduction

The overwhelming focus of acoustical wildlife recording has been on intentional vocalizations, which have long been studied using directional microphones to record focal animal sounds (Kroodsma 2005). Intentional vocalizations are also the focus of emerging technologies to monitor species presence and abundance using long-term, undirected recordings (Mennill et al. 2012). However, animals produce many incidental sounds that can offer valuable information about physiological, behavioral, and ecological processes. These sounds are typically much quieter than intentional vocalizations, but high quality recordings can be obtained by recording the sounds on or in close proximity to the animal. Recordings made on the animal also offer opportunities to obtain a spatiotemporal sample of the acoustical environment the animal experiences, and investigate the animal's responses to acoustical cues. Lastly, a continuous record of a free-ranging animal's acoustical environment will provide a complete record of their vocal activity, no matter where they roam.

Alkon et al. (1989) demonstrated the value of telemetered acoustical data for capturing the unintentional sounds associated with feeding, drinking, sniffing, walking, digging, and moving in dense vegetation in Indian crested porcupines (*Hystrix indica*)(Alkon et al. 1989). Subsequent studies have demonstrated the utility of recording the incidental sounds of foraging in penned deer (Nelson et al. 2005), and in domesticated mammals (Navon et al. 2012). Although the marine environment precludes wireless telemetry, acoustical recording tags have provided unique insights into the diving ecology of marine mammals (Burgess et al. 1998; Johnson and Tyack 2003). These audio tags represent a special case of the broader development of archival tags that sense many aspects of the host organism and its marine environment (Ropert-Coudert and Wilson 2005; Naito 2010).

Wireless telemetry of audio removes the necessity of recovering the tag, but archival recordings in the tag have several advantages. It costs much less power to store data locally than to transmit it wirelessly. Local storage can deliver much higher quality audio, with wider dynamic range. Data collection is continuous no matter how far the animal travels, removing potential limitations imposed by the communication range of the wireless system. Archival tags also eliminate the need for constant observation, and permit data to be collected continuously, even when distance, darkness, or cover obscures the animal. Moreover, tags present a logical alternative to constant observation, as the presence or approach of humans has been shown to induce both subtle physiological and overt behavioral responses in wildlife (Macarthur et al. 1982; Steen et al. 1988). One potential drawback of archival tags is the installation, requiring the animal to be captured and handled for a short period of time, which can have impacts on the animal (Delgiudice et al. 1990; Montane et al. 2002; Dickens et al. 2010).

In the present study, the primary goal was to develop on-animal acoustic collars that would allow investigation of wild mule deer (*Odocoileus hemionus*) foraging and other behaviors in relation to anthropogenic noise and spatial patterns of human disturbance. We present our design criteria and discuss their realization using a consumer audio recorder. The strengths and weaknesses of our tag are summarized to inform future tag development efforts. These tags were successfully deployed on 10 free-ranging deer. The resulting acoustic data are summarized, illustrating details of mule deer activity budgets and identifying sounds related to physiological processes and behavioral activities. These data demonstrate the potential to provide insights into species responses to anthropogenic disturbance (Francis et al. 2009) and sources of conflict with humans (Buchholz 2007).

## Methods

### Collar design

**Figure 1:**
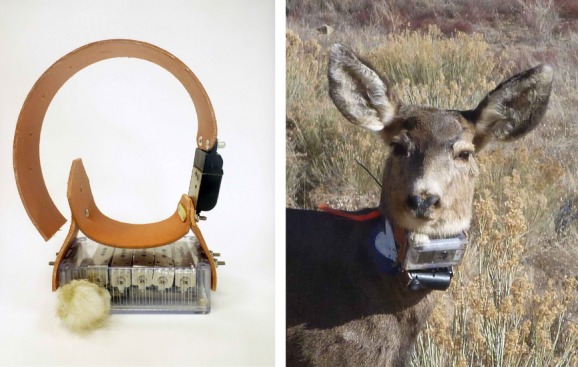
Photographs of acoustical monitoring collars during development (left) and implementation (right).

We designed and packaged ten audio recording collars (Fig. 1) for mounting on mule deer using a commercially available voice recorder (DM-420, Olympus, Center Valley, PA) powered by five lithium thionyl chloride 3.6 V AA batteries. This recorder model was selected over others because of its compact physical dimensions and low power consumption (less than 30 mA at 3 V). Although the recorder was outfitted with two internal microphones, we replaced them with one small (6 mm), high-gain external microphone capsule (Type PA3-IL, http://supercircuits.com, Austin, TX) mounted at the base of a small horn (6.19 mm throat diameter/17.95 mm mouth diameter/10.7 mm high). The horn provided mechanical protection for the microphone element, improved high frequency sensitivity, and offered a moderate amount of gain (area gain 9.24 dB above 5526 Hz) (Fristrup and Mennitt 2012). We weatherproofed the microphone and horn package with a thin sheet of plastic, which was then covered in synthetic fur fabric for wind protection. In a controlled acoustical environment (Industrial Acoustics Company, Inc., Bronx, NY), the noise floor of the complete unit was estimated to be 26.2 dBA. We configured the recorders to capture one MP3 format audio file per day to a removable 32 GB microSDHC card (because the internal memory size of the recorder was limited to 2 GB). The bitrate was set to 192 kbps for most recorders, with two units being set to 128 kbps to assess the trade-off between recording quality and file size. All recorders utilized a sampling rate of 44.1 kHz.

We fashioned the collar itself out of transmission belting material. To minimize risk of injury to the deer and to ensure prompt data recovery, each collar had a timed drop-off mechanism (Lotek, Ontario, Canada) programmed to disengage 3 weeks after the collars were mounted on the deer. A secondary detachment point (1/4″ latex tubing that degrades over time) was also instituted in case of drop-off mechanism failure. To facilitate collar recovery, ear tag transmitters (series M3600, Advanced Telemetry Systems, Isanti, MN), were attached to the collars. The audio recording collar and all associated components (including batteries, recorder, and housing) weighed approximately 280 g and cost approximately $200 each.

### Field tests

We fitted recording collars to 10 does (aged 4.5–11.5 years) that were captured using helicopter net gunning as part of an intensive radio tracking study in the Piceance Basin of northwestern Colorado (Lendrum et al. 2012). Audio recording was scheduled to begin at midnight following capture to ensure that deer had adequate time to return to home ranges and that behavioral data were collected well after physiological recovery from anesthesia. In addition to the audio recording collars, each focal deer was outfitted with a GPS collar (Model G30C, Advanced Telemetry Systems, Isanti, MN).

To corroborate behavioral observations from the acoustic collars, we performed a separate validation test on a captive mule deer at the Colorado Parks and Wildlife Foothills Wildlife Facility in Fort Collins, CO. One of the collars deployed in the field test was installed on a captive mule deer and configured to collect continuous MP3 audio files at 128 kbps. An observer simultaneously recorded the timing and sequence of several classes of behavior (browsing, ruminating, and masticating). For the purpose of the study, we define “browsing” as active intake of forage through grazing and cropping of vegetation. We categorized the intermittent regurgitations of ingesta and eructation of gas that occur during long resting or bedded periods as “ruminating.” We define “mastication” as the active and prolonged mastication of ingesta. See Figure 3 and audio recordings in supplementary material for examples of these behaviors. All protocols and procedures employed were reviewed and approved under Institutional Animal Care and Use Committee (IACUC) protocol 10-2350A.

### Acoustic analysis

Audio data were converted to WAV format (44.1 kHz sample rate, 16 bit) from their original MP3 format (128 kbps or 192 kbps). Data were then broken into 1-second segments (44,100 samples). Finally, FFT (*n* = 44,100) data points were binned into the appropriate 1/3 octave center bands to produce a 1/3 octave, 1-second Leq, which ultimately produced continuous 24 h spectrograms (Mennitt and Fristrup 2012). Spectrograms ranged in frequency from 20 Hz to 20 kHz, with 1-second time resolution. In lieu of analyzing all 3300 h of data, we used a random number generator to select 5 days of continuous audio data from one wild deer to assess within-deer variation in time spent engaged in the specified behaviors. We then randomly selected 1 day of continuous audio data from each of five wild deer to test the potential of collar-mounted microphones to assess interindividual variability in estimated time budgets.

**Figure 2:**
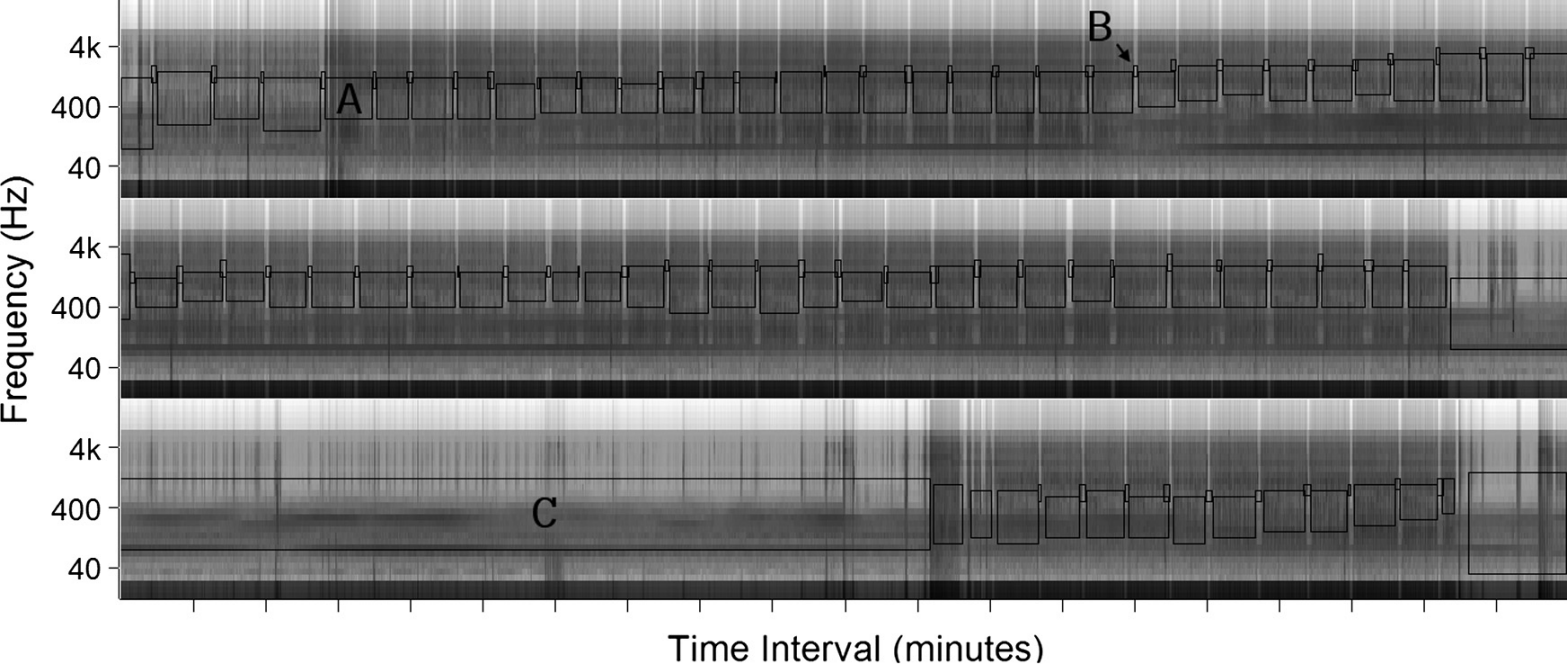
Spectrogram displaying 1 h (1:00–2:00 am) of data collected from a collar-mounted recorder plotted over three 20-minute lines. The *Y*-axis corresponds to frequency on a logarithmic scale spanning a range of 12.5 Hz–20 kHz. The shading scale represents intensity of sound level. Quiet background sound levels are assigned lighter shades and sound events are assigned darker shades. The duration of distinct classes of behavior in this spectrogram have been annotated with black boxes. This hour was dominated by 75 repeated chewing events (A) that were punctuated by an equal number of pauses (B). During the long (775 sec) event (C), the deer is largely inactive and likely bedded down.

Although much ungulate behavior can be easily distinguished by listening, these sounds can be more rapidly processed by visual review of their spectrographic signatures (Fig. 2). We used a spectrogram visualization tool created by the National Park Service Natural Sounds and Night Skies Division (Lynch et al. 2011) to identify and annotate periods associated with three components of foraging behavior: browsing, masticating, and ruminating. We confirmed the accuracy of behavioral annotations by referencing paired observations and recordings from the captive-deer validation test.

## Results

Each of the ten recording collars released on wild deer contained between 10 and 18 complete days of audio data (recordings terminated when batteries were depleted). Collar condition after the study indicated that the collars survived the harsh winter temperatures (ranging between −12°C and 2°C) and intermittent precipitation encountered during the study period without physical damage. The validation study on the captive deer revealed 100% agreement between observed behaviors and those detected by later visual analysis of the spectrograms and audio playback (i.e., there were no instances of disagreement between the two datasets).

**Figure 3:**
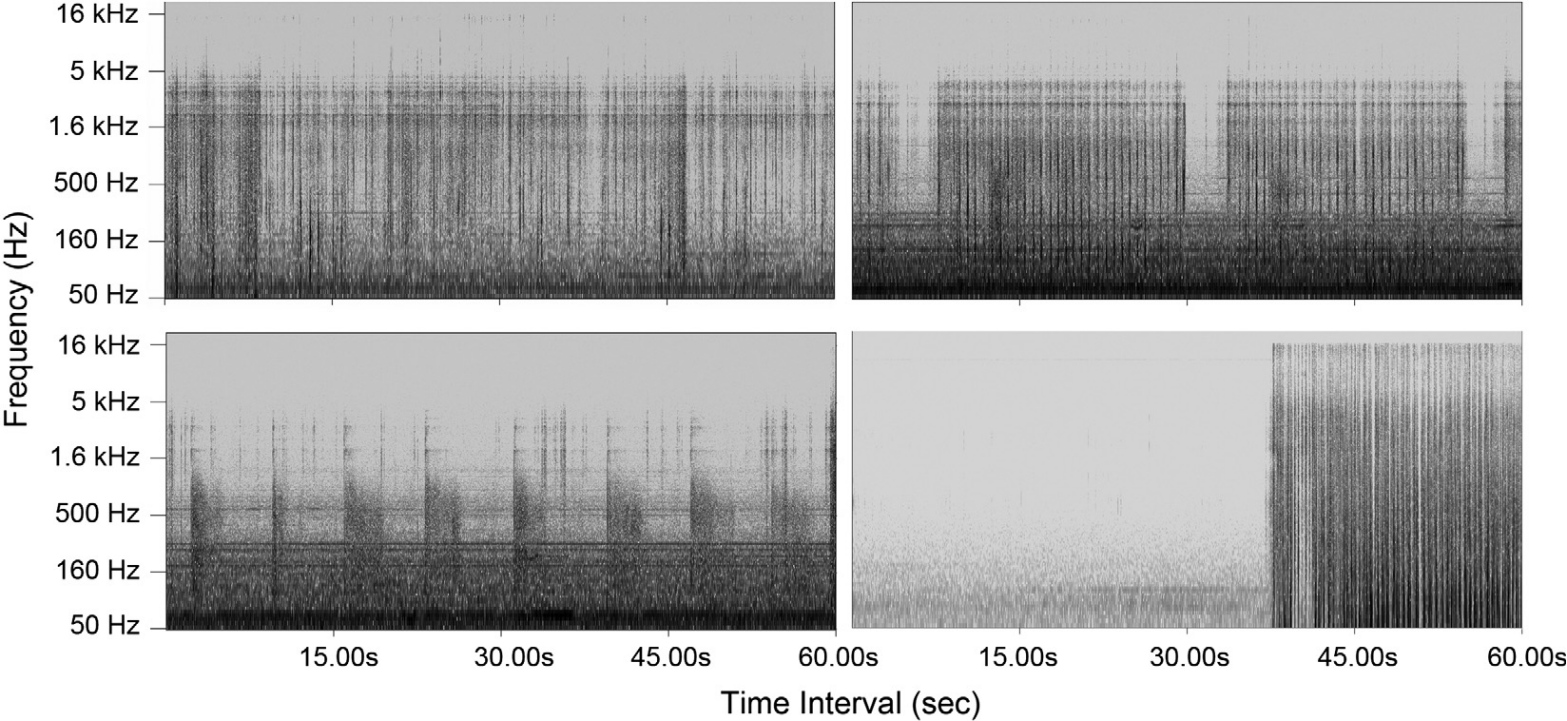
This figure shows four spectrograms each displaying 1 min of data collected from a collar-mounted recorder. Time is displayed on the *X*-axis and frequency is displayed on the *Y*-axis on a logarithmic scale. The shading scale represents intensity of sound level. Quiet background sound levels are assigned lighter shades, and sound events are assigned darker shades. Many classes of behavior possess distinctive sound signatures. Clockwise from top left, represented behaviors are: browsing (i.e., cropping of vegetation), periodic mastication separated by three pauses, respirations (eight deep breaths) during a resting period, and a startle event initiated approximately 35 sec into the recording.

Behaviors were differentiated through listening and visual review of spectrograms. Browsing could be identified by its irregular spectral pattern and was clearly distinguishable from the more rhythmic pattern created by mastication (Fig. 3). During mastication each chew was defined by a sharp vertical line in a spectrogram, which was occasionally interrupted by pauses evident as an absence of sound energy (Fig. 3). Similarly, there was a characteristic signature for respirations during prolonged resting periods, and for startle events marked by the nearly instant appearance of sound energy (Fig. 3). In cases of indistinct spectrogram signatures, corresponding audio files were played back to support accurate behavioral identification.

**Figure 4:**
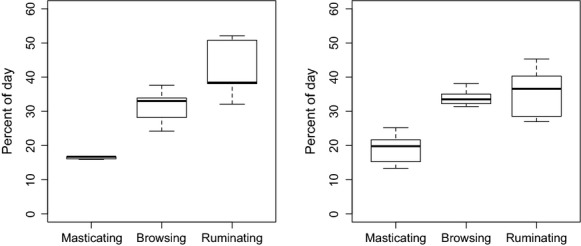
Median percent of day spent engaged in three components of foraging behavior measured for a single deer over multiple days (left panel; *n* = 5 days) and for five deer on a single day (right panel; *n* = 5 deer). Boxes indicate 25th and 75th percentiles, and whiskers span the range.

The time spent in discernible foraging activities was relatively stable for one deer across different days as well as among single days for different deer (Fig. 4). Of the three components of foraging behavior we investigated, the deer spent the least time masticating (median = 16.7% for a single deer over 5 days and 20.8% for five deer on a single day), a moderate amount of time browsing (median = 33.0% for a single deer over 5 days and 33.5% for five deer on a single day), and the most time ruminating (median = 38.4% for a single deer over 5 days and 38.3% for five deer on a single day; Fig. 2). The remainder of time was spent engaged in a variety of other behaviors. We documented frequent pausing during mastication (defined as a 3–5 sec period of complete silence during mastication bouts). Daily number of pauses during mastication for the five deer ranged from 356 to 702 with a median of 483.

**Figure 5:**
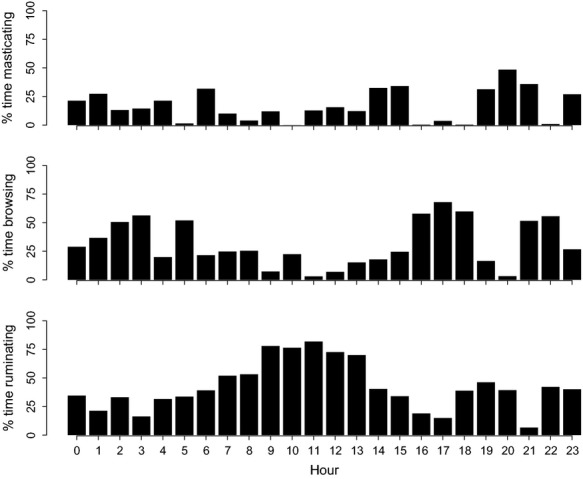
Mean hourly percent of time spent engaged in three behaviors (from top to bottom: masticating, browsing, and ruminating) measured for a single deer over 5 days.

By analyzing behavioral data on a continuous (24-hour) basis, we were able to gain insight into the diel patterns of masticating, browsing, and ruminating. As shown in Figure 5, browsing and ruminating were found to be negatively correlated (Spearman rank correlation coefficient = −0.71, *P* < 0.001). As might be expected from a crepuscular animal, browsing tended to occur in the early morning and late evening hours, while the deer was less active (ruminating) during the midday hours. Furthermore, while browsing and ruminating tended to peak at certain times of day, masticating was the most consistent behavior, in that it was observed throughout the day and night hours.

We also noted occurrences of vocalizations, grooming events, footfalls associated with movement, respirations, and startle events. The collars were sensitive enough to pick up numerous ambient environmental sounds such as bird song, coyote choruses, passing vehicles, aircraft, and other anthropogenic sound sources. Incidental environmental sounds rarely masked deer produced sounds, but these incidental environmental sounds were most easily identified when the deer were inactive (i.e., quiet).

## Discussion

We produced a durable acoustical monitoring collar capable of continuously documenting behavioral data for wild ungulates, over unlimited geographic space. Our analysis (Fig. 4) indicated that the Piceance mule deer time budgets were similar to those collected in other locations using telemetry (Kie et al. 1991). Kie et al. (1991) estimated that deer spend on average 32 ± 2.2 (SE) percent of the time feeding during intermittent browsing events, and 60 ± 2.4% resting (defined as either resting and/or ruminating) per 24-hour period (Kie et al. 1991) which is equivalent to the combination of our observations of mastication and rumination. While our findings of the study deer on winter range were consistent with previous estimates achieved through observation of ruminant time budgets (Wickstrom et al. 1984; Kie et al. 1991), they provide greater detail regarding these and other behaviors, and avoided observer effects that may arise from in situ observations. Furthermore, by sampling over continuous 24-hour time blocks, we were able to gain insight into diel patterns that could not otherwise be obtained through intermittent observation periods. Such data can allow investigation of climatic or environmentally related variation in diel activity.

In addition to documenting foraging and food processing, our acoustical data revealed periodic pauses during mastication. While the pauses clearly serve a physiological purpose, the pauses also appear to be used for acoustic vigilance. The functions of these pauses was established by visual assessment of captive deer, which appeared to use the pauses to swallow, expire gas, and then listen to their surroundings – as indicated by movements of their pinnae. We have not found any description of auditory surveillance activity in mule deer, perhaps because previous studies have focused on other cues, or occurred in open areas where vigilance is maintained by visual scanning. The Piceance Basin is characterized by relatively thick brush in the pinyon-juniper scrub ecozone and as such, visual scanning may be less effective, requiring the deer to rely on acoustical surveillance for predator detection. Additional investigation is needed to determine what temporal and acoustical conditions are likely to produce these periodic pauses, and the significance of this apparent acoustical surveillance.

### System design considerations

Fairly recent advances in audio recording technology and the advent of inexpensive, yet expansive, digital storage capacity have paved the way for the development of on-animal acoustical sensors. The collars developed for this study were constructed from economical, commercially available parts, using few production steps. Our current design required a box with dimensions generous enough to accommodate the recorder and batteries. Although the technology does not yet exist at the price point targeted, a reduction in size allowing direct mounting to a GPS collar would be ideal. Depending on the goals of the study, it should be noted that alternate positions of the horn should be considered. In our case, we positioned the horn toward the head of the deer to collect deer produced sound. The location we chose occasionally caused artifact sounds of fur rubbing against the microphone. The predominance of deer-created sound made our recordings suboptimal for recording ambient noise in the ecosystem, for which orienting the horn outwards would allow better monitoring of the environment. Although we could have used both orientations simultaneously, it would have cut our storage capacity in half.

As demonstrated, acoustical collars can provide detailed insight into fine-scale behaviors (including movement, communication, and foraging) as well as allow novel investigation of the influence of sound disturbances on ungulates. As shown in Table 1, behavioral data produced by acoustical collars can clarify species habitat needs and nutritional ecology (Nelson et al. 2005). This technology can also be used to study foraging behavior (including intake and efficiency) of wild or domestic animals (Laca & WallisDeVries 2000), and to parameterize activity budget data and energetic modeling on a finer scale than has been produced before. On-animal audio recording devices also have the potential to advance communication studies in vocal species and to inform stimulus–response studies on a landscape scale. Finally, for acoustic ecologists interested in the effects of noise on wildlife, this type of acoustical monitoring can provide accurate measurements of the intensity of noise stimulus presented to the individual at any given time. Recent study has shown that MP3 audio can be translated into calibrated sound pressure levels (Mennitt and Fristrup 2012).

**Table 1:** For each study type, the audible behaviors that could be captured by an on-animal acoustical monitoring device

Study type	Recorded sounds (behavioral or environmental)
Time budget	Foraging, resting, grooming, walking
Communication	Vocalizing (expressed and heard)
Reproduction	Courtship, male contests, mating, birthing
Movement	Footfalls
Nutrition	Cropping rates, masticating, ruminating
Physiology	Respiring, excreting
Phenology	Timing of initiation of specific behaviors
Acoustic ecology	Audible ambient noise
Interspecific interactions (predation events)	Predator vocalizations, chasing, and killing
Impacts from human disturbance	Intensity of stimulus, reacting through startle events

Along with many benefits, acoustical monitoring does have limitations. While it saves vast amounts of time in field observations, it also generates large datasets, which can be daunting to process. However, numerous automatic processing software packages exist (such as Raven, XBAT, SongScope, Ishmael, and many others) to help users identify signals of interest. Even so, acoustical datasets may require concurrent observational periods to confirm proper identification of ambiguous sound signals. In addition, while on-animal tags reduce the observer effect, they also require capture for installation, which may introduce both acute and chronic stress into the behavioral study system. Despite these disadvantages, acoustical monitoring remains an inexpensive, adaptable, and accurate method for recording animal behavior. Moreover, the training data we produced with manual spectrogram annotation has the potential to inform automated detection of certain behaviors across species.

## Supporting Information

**Audio S1.** 60 second recording of browsing behaviour.

**Audio S2.** 60 second recording of masticating behaviour.

**Audio S3.** 60 second recording of respirations during resting period.

**Audio S4.** 60 second recording of startle event.

## References

[b1] Alkon PU, Cohen Y, Jordan PA (1989). Towards an acoustic biotelemetry system for animal behavior studies. J. Wildl. Manage.

[b2] Buchholz R (2007). Behavioural biology: an effective and relevant conservation tool. Trends Ecol. Evol.

[b3] Burgess WC, Tyack PL, Costa BJ, Le Boeuf DP (1998). A programmable acoustic recording tag and first results from free-ranging northern elephant seals. Deep Sea Res. Part II.

[b4] Delgiudice GD, Kunkel KE, Mech LD, Seal US (1990). Minimizing capture-related stress on white-tailed deer with a capture collar. J. Wildl. Manage.

[b5] Dickens MJ, Delehanty DJ, Romero ML (2010). Stress: an inevitable component of animal translocation. Biol. Conserv.

[b6] Francis CD, Ortega CP, Cruz A (2009). Noise pollution changes avian communities and species interactions. Curr. Biol.

[b7] Fristrup K, Mennitt D (2012). Bioacoustical monitoring in terrestrial environments. Acoust. Today.

[b8] Johnson MP, Tyack PL (2003). A digital acoustic recording tag for measuring the response of wild marine mammals to sound. IEEE J. Oceanic Eng.

[b9] Kie JG, Evans CJ, Loft ER, Menke JW (1991). Foraging behavior by mule deer: the influence of cattle grazing. J. Wildl. Manage.

[b10] Kroodsma D (2005). The singing life of birds: the art and science of listening to birdsong.

[b11] Laca & WallisDeVries (2000). Acoustic measurement of intake and grazing behaviour of cattle. Grass Forage Sci.

[b12] Lendrum PE, Anderson CRJ, Long RA, Kie JG, Bowyer RT (2012). Habitat selection by mule deer during migration: effects of landscape structure and natural-gas development. Ecosphere.

[b13] Lynch E, Joyce D, Fristrup K (2011). An assessment of noise audibility and sound levels in US National Parks. Landscape Ecol.

[b14] Macarthur RA, Geist V, Johnston RH (1982). Cardiac and behavioral responses of mountain sheep to human distubance. J. Wildl. Manage.

[b15] Mennill DJ, Battiston M, Wilson DR, Foote JR, Doucet SM (2012). Field test of an affordable, portable, wireless microphone array for spatial monitoring of animal ecology and behaviour. Methods Ecol. Evol.

[b16] Mennitt DJ, Fristrup KM (2012). Obtaining calibrated sound pressure levels from consumer digital audio recorders. Appl. Acoust.

[b17] Montane J, Marco I, Manteca X, Lopez J, Lavin S (2002). Delayed acute capture myopathy in three roe deer. J. Vet. Med. Ser. A.

[b18] Naito Y (2010). What is “bio-logging”?. Aquat. Mamm.

[b19] Navon S, Mizrach A, Hetzroni A, Ungar ED (2012). Automatic recognition of jaw movements in free-ranging cattle, goats and sheep, using acoustic monitoring. Biosyst. Eng.

[b20] Nelson DE, Alkon PU, Krausman PR (2005). Using acoustic telemetry to monitor foraging by penned mule deer. Wildl. Soc. Bull.

[b21] Ropert-Coudert Y, Wilson RP (2005). Trends and perspectives in animal-attached remote sensing. Front. Ecol. Environ.

[b22] Steen JB, Gabrielsen GW, Kanwisher JW (1988). Physiological-aspects of freezing behavior in willow ptarmigan hens. Acta Physiol. Scand.

[b23] Wickstrom ML, Robbins CT, Hanley TA, Spalinger DE, Parish SM (1984). Food Intake and foraging energetics of elk and mule deer. J. Wildl. Manage.

